# The Developmental Eye Movement Test as a Diagnostic Aid in Cerebral Visual Impairment

**DOI:** 10.3389/fnhum.2021.732927

**Published:** 2021-10-28

**Authors:** Nouk Tanke, Annemiek D. Barsingerhorn, Jeroen Goossens, F. Nienke Boonstra

**Affiliations:** ^1^Department of Cognitive Neuroscience, Donders Institute for Brain, Cognition and Behavior, Radboud University Medical Centre, Nijmegen, Netherlands; ^2^Department of Biophysics, Donders Institute for Brain, Cognition and Behavior, Radboud University, Nijmegen, Netherlands; ^3^Royal Dutch Visio, National Foundation for the Visually Impaired and Blind, Nijmegen, Netherlands; ^4^Behavioral Science Institute, Radboud University, Nijmegen, Netherlands

**Keywords:** cerebral visual impairment (CVI), visual impairment (VI), visual processing speed, diagnostic, developmental eye movement test (DEM)

## Abstract

The symptoms that characterize children with cerebral visual impairments (CVI) are diverse, ranging from extensive behavioral or physical disabilities to subtle changes that can easily be missed. A correct diagnosis of CVI is therefore difficult to make, but having a wide variety of tests available can be helpful. This study aims to determine if the developmental eye movement test (DEM) can be one of those tests. In this test, a fixed set of numbers has to be read aloud, first in vertical columns and then in horizontal lines. In order to measure differences between children with CVI compared to normally sighted age-matched controls and children with a visual impairment (VI), we determined DEM times, crowding intensities and the reaction time to a large visual stimulus for all three groups. We found that children with CVI or VI need significantly more time to read the DEM numbers than age-matched controls. Additionally, children with CVI need more time than children with VI to read the horizontal DEM, but not the vertical DEM. We also found a significant difference between the children with CVI and the other two groups in the relationship between horizontal DEM performance and crowding intensity. However, for the relationship between DEM performance and visual detection time, no group-differences were found. We conclude that the DEM can be a useful addition in the diagnosis of CVI, especially in combination with information about crowding.

## Introduction

The Developmental Eye Movement test (DEM) is a number naming test that was originally designed to measure oculomotor deficiencies without expensive equipment (Garzia et al., 1990). The first two subtests measure the time that a child needs to read two columns of numbers from top to bottom. The third subtest records the time needed to read sixteen rows of numbers from left to right. Although previous studies indicate that comparing the horizontal and vertical DEM scores is not a good method to detect oculomotor deficiencies (Ayton et al., 2009; Tanke et al., 2021), the DEM does reflect clinically relevant factors. For instance, DEM performance relates to the level of academic performance (Garzia et al., 1990; Wood et al., 2018; Hopkins et al., 2019), reading rate (Northway, 2003; Palomo-Alvarez and Puell, 2009; Facchin et al., 2011; Serdjukova et al., 2016), and speed of visual processing (Ayton et al., 2009; Tanke et al., 2021).

Slower visual processing often occurs in children with cerebral visual impairments (CVI) (Kooiker et al., 2016; Barsingerhorn et al., 2018b). CVI is a visual impairment that is caused by malfunctions in the central visual pathways due to pre, post or perinatal damage to the brain or caused by congenital abnormalities which can lead to visual impairments like difficulties with contrast, depth or recognizing objects (for reviews, see Philip and Dutton, 2014; Lueck et al., 2019). CVI impairments are widely variable and can sometimes be subtle and difficult to detect (Lowery et al., 2006). For example, the visual acuity of children with CVI can range from complete blindness to normal vision. Yet, even children with CVI with a normal visual acuity often show exacerbated visual crowding (van Genderen et al., 2012), a difficulty identifying visual information when it is closely surrounded by visual flankers (Bouma, 1970; Huurneman et al., 2012a). It is because of these wide-ranging features that a wide variety of diagnostic tools is required. This study aims to determine if the DEM can be one of those diagnostic aids for children with CVI.

Previous work has shown that, for normally sighted (NS) children, the DEM scores are positively correlated to fixation duration and visual processing speed (Tanke et al., 2021). However, when looking at cartoons, children with CVI use shorter fixation durations compared to controls and children with visual impairments (VI), but they direct their gaze to a larger area (Kooiker et al., 2016), suggesting that the fixation control of children with CVI is not optimal. Additionally, adults with CVI show a more diffuse search pattern than controls when visual information becomes more crowded (Bennett et al., 2019). During the horizontal DEM, the numbers are inconsistently spaced to minimize automaticity (Garzia et al., 1990). This inconsistent spacing can be challenging for children who use sub-optimal search patterns. We therefore studied the differences in DEM scores and visual information processing between NS children, children with CVI and children with VI. DEM performance, in combination with information about visual processing speed and crowding, can provide valuable information for the diagnosis of children with CVI.

## Methods

### Participants

A total number of 158 children aged 5–17 years were recruited. All the children had a visual acuity better than 1.3 LogMAR. For NS children (*n* = 96, 9.4 ± 2.0 years) and children with VI (*n* = 33, 9.0 ± 2.4 years), inclusion criteria were normal birth weight (> 2,500 g), birth at term (> 36 weeks), no perinatal complications and normal development. NS children had a linear distant visual acuity of 0.1 logMAR or better, VI children had a visual acuity worse than 0.1 LogMAR. The only inclusion criterium for the children with CVI (*n* = 30, 9.2 ± 1.6 years) was having the diagnosis of CVI. The diagnosis of CVI was made by ophthalmologists of Bartiméus institute or Royal Dutch Visio, Dutch institutes for the rehabilitation of the visually impaired. After 2019, the Dutch CVI guidelines were applied to obtain the diagnosis (Federatie Medisch Specialisten, 2019). Children with glasses wore them during all tests. For NS children, testing occurred at the children’s primary schools. Children with VI and CVI were recruited from Bartiméus (VI; *n* = 28, CVI; *n* = 16) or the Royal Dutch Visio (VI; *n* = 5, CVI; *n* = 14, for details see Barsingerhorn et al., 2018b; Supplementary Table 1).

Informed consent was obtained from the parents of all participants. The study was approved by the local ethics committee Commissie Mensgebonden Onderzoek regio Arnhem-Nijmegen, Netherlands (protocol NL48708.091.14), and conducted according to the principles of the Declaration of Helsinki.

### Ophthalmologic Examination

The Freiburg visual acuity test (Bach, 1996) was used to determine the distal visual acuity. For NS children, the uncrowded chart used a fixed inter-letter spacing of at least 30 arcmin, while crowded visual acuity was measured with spacing of 2.6 arcmin. For the children with VI or CVI, crowding was determined binocularly with the LEA version of the *C*-test. The LEA test consisted of the same uncrowded and crowded chart versions as the *C*-test but presented symbols in a larger size range of 0.3–1.7 logMAR.

Crowding intensity was defined as the difference between crowded and uncrowded visual acuities in logMAR (Huurneman et al., 2016c).

### Developmental Eye Movement Test

**FIGURE 1:**
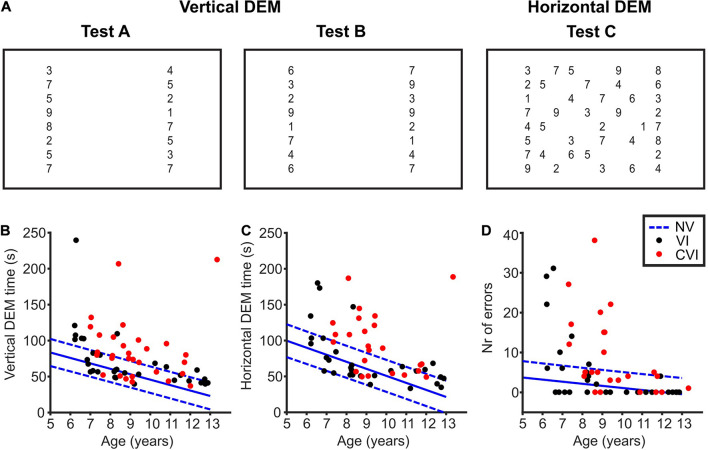
The developmental eye movement test (DEM). **(A)** Overview of the DEM test. Numbers not drawn to scale. Adopted from Tanke et al. (2021). **(B)** Total time needed to read the numbers of the vertical DEM (test A + B) for children with VI (black dots) and CVI (red dots). In blue, the mean (solid line) vertical DEM times of NS children and the corresponding 90% prediction intervals indicated by the 5th and 95th percentile (dashed lines) of the duration distributions. **(C)** Same as for **(B)**, but for the error-adjusted horizontal DEM time (test C). **(D)** Same as for **(B)**, but for the number of errors made during the horizontal DEM.

The DEM (Figure 1A) consisted of high contrast numbers of 4.9 mm in height (LogMAR 0.71, similar to the paper version of the DEM) that had to be read aloud. For each subtest of the DEM, the numbers were shown all at once. Children first practiced with a DEM pre-test to familiarize them with the task, and to make sure that they could read the numbers. The pre-test was a shortened version of each DEM subtest with randomized ordering of the numbers. Then, children had to name the numbers of test A from top to bottom, one column at a time, as quickly as possible. All the numbers of a subtest appeared on the computer screen as soon as the experimenter pressed the space bar and disappeared when the experimenter pressed the space bar again as soon as the child had read the last number. These start and stop moments were recorded by the software. Test A was followed by test B, which is similar to A but with the numbers in a different order.

The numbers of test C had to be named row by row from left to right, starting at the top left. Horizontal time was taken as the total time to name the first to the last number of test C. For the full list of numbers used and details concerning number spacing and number size see Garzia et al. (1990) and Tanke et al. (2021).

### Visual Detection Task

The children also performed a visual detection task (VDT) at the same distance as the DEM to measure the time children needed to respond to a supra-threshold stimulus. In the VDT (20 trials), they had to press a mouse button as soon as they saw the visual stimulus (a large high-contrast black letter “O,” for details see Barsingerhorn et al., 2018a).

### Equipment

The stimulus software used at the schools and Bartiméus was written in Matlab (version 2013b) using the Psychophysics Toolbox (version 3.0.12; Kleiner et al., 2007). At Royal Dutch Visio, the stimulus software was written in Python using PsychoPy3 (version 2020.2.10; Peirce et al., 2019). In both versions of the software, stimulus timing and button presses were recorded and stored at millisecond precision. The visual stimuli were presented on a 23-inch LCD screen (Dell, Inc., 1,920 × 1,200 pixels).

### Procedure

To assess the visual acuity binocularly, children first participated in the Freiburg visual acuity test (Bach, 1996) administered digitally at 5 meters distance. Then, a paper version of the LEA test was administered at 40-cm viewing distance. Secondly, the computer screen was moved to ∼65 cm viewing distance for the DEM and the VDT.

### Data Analyses

The offline analysis was performed and images were created using Matlab (version 2020b).

#### DEM

Total vertical time was taken as test A + test B. If only test A was completed, vertical time was taken as 2 times test A (Tanke et al., 2021). Time to complete test C was adjusted for omissions and additions (Richman and Garzia, 1987). Repeating a whole line counted as five addition errors. Skipping one-line counts as two omission errors. The time for test A and B was not adjusted for errors because of the limited number of errors made during those tests. The number of errors was determined by adding the number of omissions and additions in test *C*.


AdjustedtimetestC=TimetestC[80/(80-omissions+additions)]*

#### VDT

Mean reaction times were computed after removing atypically long or short reaction times. Trials were excluded if the reaction time deviated more than three times the median absolute deviation from the median after discarding reaction times < 0.1 s.

### Statistical Analyses

Correlation coefficients were calculated using Pearson’s correlation coefficient, and Pearson’s linear partial correlation with age as a confounding variable. Multiple linear regression with age as a co-variable was used to test average differences in DEM times between groups, using group as a categorical variable (model: *DEM* ∼ *group* + *age*, Wilkinson notation). Subsequently, we included either the reaction time measured in the VDT or the crowding intensity in the regression to test whether these factors could account for any additional variability between participants. The power (1–β) of these regression models to detect medium-size effects (*f*^2^ = 0.15) at a significance level (α) of 0.05 was > 0.95 (Faul et al., 2007, 2009). Furthermore, a Kolmogorov-Smirnov test showed that both the vertical DEM scores and the horizontal DEM scores were normally distributed. To assess the impact of outliers, robust linear regressions were performed too, but since the results were similar to standard linear regression with equal weights for all data points, they are not reported in this manuscript.

## Results

Children were asked to read the numbers of the DEM aloud (Figure 1A). All the children participated in both the vertical and the horizontal DEM. However, the horizontal DEM was too difficult for a small number of children (NS, 2/96; VI, 2/33; CVI, 6/30). These children did not read the numbers row by row, but skipped from one row to another on numerous occasions, making it impossible for the experimenter to follow which number was read from which location. We therefore excluded the horizontal DEM scores of these 10 participants. For the other 149 children, scores were correctly documented and the horizontal DEM time was adjusted for the number of errors made (see section “Methods” for details).

### Developmental Eye Movement Test Scores and Age

When looking at Figures 1B,C, it is implicated that DEM times get better with age. For both the vertical DEM and the horizontal DEM, the relationship between DEM and age for the CVI and VI groups did not differ significantly from the NS children [vertical DEM; VI; *t*(126) = 1.06, *p* = 0.29, CVI; *t*(123) = −0.59, *p* = 0.55, horizontal DEM; VI; *t*(122) = 0.36, *p* = 0.72, CVI; *t*(115) = 1.50, *p* = 0.14, DEM∼age^∗^group]. For all the groups taken together, the slope of the vertical DEM was −6.61 ± 0.85 s/year (*p* < 0.001) and the slope of the horizontal DEM was −9.80 ± 1.30 s/year (*p* < 0.001). This shows that, indeed, DEM performance is age-dependent for all three groups of children. Therefore, age was included in all the regression analyses in this study.

### Group Differences in Developmental Eye Movement Test Scores

NS children needed on average 50.3 s (95% CI: 46.6–54.1) to read the numbers of the vertical DEM, and 56.1 s (95% CI: 51.4–60.8) for the horizontal DEM. Children with VI, on the other hand, needed significantly more time [Figures 1B,C, vertical DEM; 20.2 s longer (95% CI: 10.3−30.1), *t*(126) = 3.44, *p* < 0.001, horizontal DEM; 18.1 s longer (95% CI: 6.8−29.3), *t*(122) = 3.05, *p* = 0.003, linear regression]. Likewise, children with CVI needed more time than NS children [vertical DEM; 35.4 s longer (95% CI: 24.7−46.1), *t*(123) = 6.51, *p* < 0.001, horizontal DEM; 40.2 s longer (95% CI: 27.7−52.7), *t*(115) = 7.34, *p* < 0.001, linear regression]. In total, 11/33 children with VI and 18/30 children with CVI scored above the 95th percentile of NS children for the vertical DEM. For the horizontal DEM, that was the case for 8/31 children with VI and 15/24 children with CVI. No significant differences were found in vertical DEM scores between children with VI and CVI [15.2 s shorter for VI (95% CI: −4.8−35.2), *t*(60) = 1.73, *p* = 0.09]. However, children with CVI did need more time than children with VI to read the numbers of the horizontal DEM [22.2 s longer (95% CI: 0.7−43.7), *t*(52) = 2.53, *p* = 0.015, linear regression].

This difference between the two patient groups was also seen in the number of errors made during the horizontal DEM. Children with CVI made significantly more errors than children with VI [Figure 1D, 4.2 more errors (95% CI:−0.8−9.1), *t*(52) = 9.39, *p* < 0.001, Poisson regression]. NS children made on average 1.4 errors (95% CI: 0.9−1.9), which was significantly less than children with VI and CVI [VI; 3.4 more errors (95% CI: 1.5−5.3), *t*(122) = 8.18, *p* < 0.001, CVI; 7.6 more errors (95% CI: 5.3−9.8), *t*(115) = 18.13, *p* < 0.001, Poisson regression]. A total number of 6/31 children with VI and 10/24 children with CVI scored above the 95th percentile of the control group.

### Visual Detection Time

All the children in this study also participated in a simple VDT where a button had to be pressed as soon as a large circle appeared on a computer screen (Barsingerhorn et al., 2018a). An increased reaction time in the VDT indicates that the child needs more time to respond to visual information. NS children took, on average, 347 ms (95% CI: 329−366) to respond to the VDT stimulus. Children with VI needed, on average, 84.4 ms (95% CI: 46.3−122.4) more to respond to the VDT, and children with CVI 187.7 ms (95% CI: 143.7−231.6) more. As described previously, vertical and horizontal DEM times were strongly correlated to visual processing speed for NS children (Tanke et al., 2021).

**FIGURE 2:**
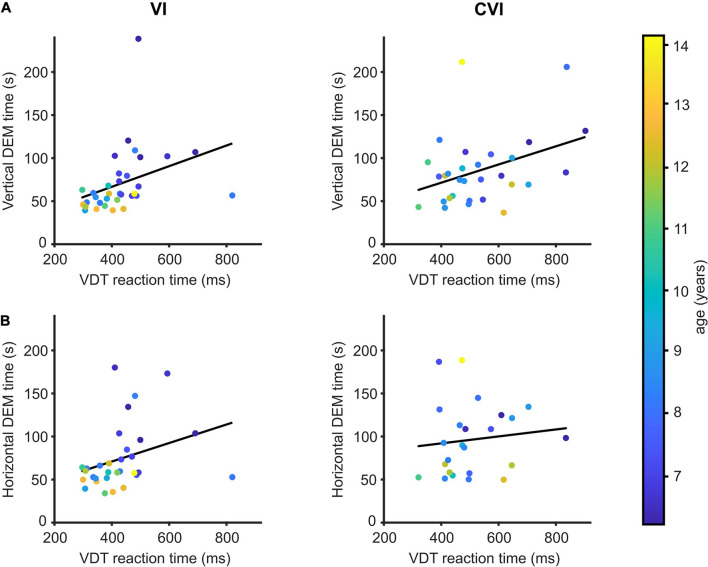
DEM performance in relation to visual detection time. **(A)** Mean reaction time to detect a large visual stimulus compared to the time needed to perform the vertical DEM for children with VI (left) and children with CVI (right). Regression lines in black. Data points are color-coded by age of the child. **(B)** Same as **(A)**, but for the horizontal DEM.

Figure 2 shows the relationships between DEM performance and VDT for the CVI and VI groups. No significant differences were found between the two patient groups for the regression between VDT and DEM performance adjusted for the effect of age [vertical DEM; *t*(58) = 0.07, *p* = 0.95, horizontal DEM; *t*(50) = 0.26, *p* = 0.80, DEM∼VDT^∗^group + age]. Therefore, the two groups were pooled together in the analysis. A significant relationship was found between VDT and vertical DEM time [*t*(60) = 2.74, *r* = 0.33, *p* = 0.008, DEM∼VDT + age] but not between VDT and the horizontal DEM [*t*(52) = 1.54, *r* = 0.21, *p* = 0.13].

### Crowding Intensity

Additional to the VDT, the crowding intensity was determined for most but not all of the participating children (NS; 91/96, VI; 24/33, CVI; 28/30). Children with VI had a mean crowding intensity of 0.18 LogMAR (95% CI: 0.12–0.24) compared to 0.16 LogMAR (95% CI: 0.12–0.21) for the children with CVI. NS children had an average crowding intensity of 0.06 LogMAR (95% CI: 0.04–0.08), and vertical and horizontal DEM times were strongly correlated to the crowding intensity (data not shown; vertical DEM; *r* = 0.41, *p* < 0.001, horizontal DEM; *r* = 0.42, *p* < 0.001). However, since both DEM performance (Tanke et al., 2021) and crowding (Huurneman et al., 2012a) are age-dependent in NS children, the correlation between DEM performance and crowding intensity was lost when age was added as a covariate (correlation after adjusting for age: vertical DEM; *r* = 0.15, *p* = 0.15, horizontal DEM; *r* = 0.19, *p* = 0.07).

**FIGURE 3:**
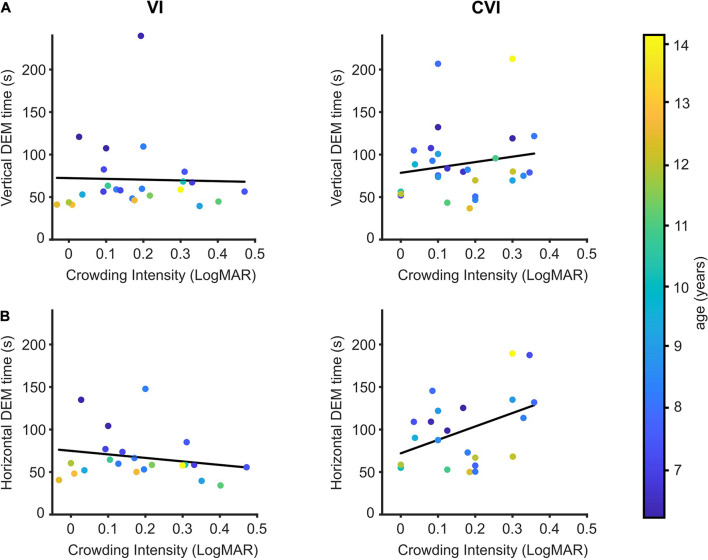
DEM performance in relation to crowding intensity. **(A)** Crowding intensity compared to the time needed to perform the vertical DEM for children with VI (left) and children with CVI (right). Regression lines in black. Data points are color-coded by age of the child. **(B)** Same as **(A)**, but for the horizontal DEM.

No significant differences were found between the VI and CVI groups for the regression between crowding intensity and vertical DEM performance adjusted for the effect of age [Figure 3A, *t*(47) = 1.01, *p* = 0.32, DEM∼crowding^∗^group + age]. With the two patient groups pooled together, no significant relationship was found between crowding intensity and vertical DEM performance [*t*(49) = 0.38, *p* = 0.71, DEM∼crowding + age]. However, for the relationship between horizontal DEM performance and crowding intensity we did find a significant difference between the children with CVI and the children with VI [Figure 3B *t*(39) = 2.77, *p* = 0.008, DEM∼crowding^∗^group + age]. A significant relationship between crowding intensity and horizontal DEM was found for children with CVI [*t*(19) = 2.20, *r* = 0.45, *p* = 0.04], but not for the children with VI [*t*(19) = 1.47, *r* = −0.32, *p* = 0.16, DEM∼crowding + age].

## Discussion

Our results show that children with CVI and VI need significantly more time to read the numbers of the vertical and the horizontal DEM than age-matched controls. Additionally, children with CVI need more time to read the horizontal DEM than children with VI. We also explored whether visual detection time and visual crowding could explain some of the variability in DEM scores of the patients, and whether these factors played a different role in children with VI compared to children with CVI. We found that individual differences in crowding intensity, but not in visual detection time, explained part of the variation in horizontal DEM scores.

### Developmental Eye Movement Test Scores and Age

Vertical and horizontal DEM times improve with age for NS children. The results for the CVI and VI groups were not significantly different from those in the NS group. However, within the group of children with CVI, the correlation between age and DEM performance was not statistically significant (vertical DEM; *r* = −0.02, *p* = 0.91, horizontal DEM; *r* = −0.18, *p* = 0.39). Perhaps, this is due to the relatively small group size. Another possibility is that the developmental age of children with CVI does not match their biological age, and that DEM performance would be better explained by the children’s developmental age. Since we could only include children who can read numbers, children with severe developmental impairments were not represented in this dataset. Although children with VI have ocular deficiencies that cause a decreased visual acuity (for details, see Supplementary Table 1), their birthweight and development is suspected to be normal (1/33 showed signs of developmental delay). Ten out of the thirty children with CVI, on the other hand, were suspected to show a developmental delay, making the exact developmental age of these children difficult to determine. It is therefore possible that our results underestimate the relation between DEM performance and age.

### Developmental Eye Movement Test Performance Differences Between Groups

Children with VI or CVI need more time than NS children to read the numbers of both the vertical and the horizontal DEM. Especially the horizontal DEM was challenging for some of the children with VI or CVI. On average, children with CVI made significantly more errors on this subtest than children with VI. In fact, we may have underestimated this difference: twenty percent (6/30) of the children with CVI were unable to correctly participate in the horizontal DEM even though they had no problems reading the numbers of the vertical DEM. By comparison, only 6% (2/33) of the children with VI and 2% (2/96) of the NS children had so much trouble with the horizontal DEM that their errors could not be scored correctly. This fact alone indicates difficulties for CVI children in recognizing complicating information. DEM scores can be affected by a variety of factors, like academic performance and number naming. For example, two boys with CVI had difficulties recognizing the number seven; they had to count up from four every time. Another possible factor that can influence DEM times is oculomotor behavior; 20/33 children with VI and 8/30 children with CVI experienced some degree of nystagmus (Supplementary Table 1). However, reading speed can be nearly normal for people with infantile nystagmus (Barot et al., 2013; Dysli and Abegg, 2016; Huurneman et al., 2016b). Children with CVI need more time to read the horizontal DEM than children with VI. This effect could be due to differences in visual acuity or oculomotor problems. However, oculomotor problems, especially nystagmus, were most frequent in the VI group (Supplementary Table 1) and the acuity of children with VI was, on average, worse than that of children with CVI (VI; 0.38 ± 0.23 LogMAR, CVI; 0.17 ± 0.25 LogMAR). Therefore, one would expect poorer performance of the children with VI on the DEM if reduced visual acuity and oculomotor problems were the main predictors of reduced DEM performance. Additionally, no difference was found in vertical DEM performance between the two groups, which should be equally affected by acuity. Finally, no significant correlations were found between visual acuity and DEM performance (vertical DEM; VI, *r* = −0.07, *p* = 0.69, CVI, *r* = −0.17, *p* = 0.38, horizontal DEM; VI, *r* = 0.10, *p* = 0.59, CVI, *r* = −0.34, *p* = 0.10), indicating that visual acuity alone did not affect DEM performance in our sample. One possible reason for the difference found in horizontal DEM performance between patient groups might be that children with CVI tend to use sub-optimal search patterns (Kooiker et al., 2016; Bennett et al., 2019), which could likely affect horizontal DEM time. Additionally, children with CVI show significantly shorter fixation durations than VI children (Kooiker et al., 2016), which might lead to problems with keeping track of where the last number was found, especially when the next number is not always present in the expected location.

### The Influence of Visual Detection Time

DEM performance is correlated to visual processing speed (Ayton et al., 2009; Tanke et al., 2021) and visual processing speed is often reduced in children with VI and CVI (Kooiker et al., 2016; Barsingerhorn et al., 2018b). However, for the relationship between DEM performance and visual processing speed, no significant differences were found between the two patient groups. This would indicate that information about DEM performance in combination with reaction times to a visual stimulus, is not sufficient to clinically differentiate CVI from other visual impairments. When regarding the children with VI and CVI as one group, the mean reaction time to the visual stimulus correlates to vertical DEM performance, but not to horizontal DEM performance. Maybe, the reason why this relationship with visual detection time is only found for the vertical DEM is because the vertical DEM is easier and less affected by additional factors like visual search and oculomotor impairments than the horizontal DEM. Note also that the VDT on its own is not a direct measure of visual processing speed, as the reaction time can also increase as a result of impaired motor skills. Twelve children in the patient groups suffered from a light motor impairment (CVI, *n* = 10, VI; *n* = 2), we therefore recommend combining DEM performance with a task more specifically designed to measure visual processing speed (see for example Barsingerhorn et al., 2018a).

### The Influence of Visual Crowding

Both the horizontal DEM numbers, as well as the numbers of the vertical DEM are spaced too far apart to be considered crowded (Huurneman et al., 2012b), indicating that difficulties with visual crowding are unlikely to directly affect DEM performance. Nevertheless, we found a significant correlation between the crowding intensity and horizontal DEM performance in children with CVI, and this correlation was significantly different from the children with VI. Perhaps, this difference was found because crowding limits reading performance (Bricolo et al., 2015; Gori and Facoetti, 2015; Joo et al., 2018), and both reading performance and crowding are linked to plasticity of the visual system (Huurneman et al., 2016a,b; Huurneman and Goossens, 2021).

## Conclusion

The DEM is a useful addition to visual function tests to diagnose CVI especially in combination with knowledge about the crowding intensity. If a child shows both a longer horizontal DEM performance and a high crowding intensity, this may be an additional pointer to the diagnosis CVI. A slower DEM performance can be an indication that the child is at a disadvantage concerning visual processing speed, but in daily life, this may be compensated for by allowing extra time and by reducing visual clutter.

## Data Availability Statement

The raw data supporting the conclusions of this article will be made available by the authors, without undue reservation.

## Ethics Statement

The studies involving human participants were reviewed and approved by Commissie Mensgebonden Onderzoek regio Arnhem-Nijmegen, Netherlands. Written informed consent to participate in this study was provided by the participants’ legal guardian/next of kin.

## Author Contributions

AB, JG, and FB designed the experiment. AB performed the experiments at schools and Bartimeus. NT performed the experiments at Royal Dutch Visio. NT analyzed the data and wrote the article with input from AB, JG, and FB. All authors contributed to the article and approved the submitted version.

## Conflict of Interest

The authors declare that the research was conducted in the absence of any commercial or financial relationships that could be construed as a potential conflict of interest.

## Publisher’s Note

All claims expressed in this article are solely those of the authors and do not necessarily represent those of their affiliated organizations, or those of the publisher, the editors and the reviewers. Any product that may be evaluated in this article, or claim that may be made by its manufacturer, is not guaranteed or endorsed by the publisher.
