# Supplementary material for: The Developmental Eye Movement Test as a Diagnostic Aid in Cerebral Visual Impairment
Source: Front Hum Neurosci. 2021 Oct 28;15:732927. doi: 10.3389/fnhum.2021.732927 (PMC8582923; doi:10.3389/fnhum.2021.732927)
Supplement: Supplementary file 1 [file Data_Sheet_1.PDF]

*Supplementary Table 1. Clinical characteristics of the children with VI and CVI*

Participant number (Pp), age, visual acuity in LogMAR (VA), group, diagnosis, and the presence of motor impairment, mental delay, strabismus and nystagmus
(+: manifest, +/-: latent, -: absent).

| **Pp** | **Age** | **VA** | **Group** | **Diagnosis** | **Motor imp** | **Mental delay** | **Strabismus** | **Nystagmus** |
|---|---|---|---|---|---|---|---|---|
| **1** | 8 | 0.3 | VI | Optic nerve hypoplasia | - | - | +/- | - |
| **2** | 6 | 0.3 | VI | Congenital stationary night blindness (CSNB) | - | - | - | - |
| **3** | 6 | 0.4 | VI | Congenital stationary night blindness (CSNB) | - | - | + | - |
| **4** | 8 | 0.3 | VI | Infantile nystagmus syndrome | - | - | - | + |
| **5** | 9 | 0.1 | VI | Hypermetropia | - | - | + | - |
| **6** | 6 | 0.3 | VI | Albinism | - | - | - | +/- |
| **7** | 8 | 0.2 | VI | Macular hypoplasia | - | - | + | + |
| **8** | 8 | 0.3 | VI | Infantile nystagmus syndrome | - | - | + | + |
| **9** | 12 | 1.2 | VI | Congenital stationary night blindness (CSNB) | - | - | - | + |
| **10** | 12 | 0.3 | VI | Congenital stationary night blindness (CSNB) | - | - | - | - |
| **11** | 6 | 0.5 | VI | Albinism | - | - | +/- | + |
| **12** | 6 | 0.3 | VI | Congenital stationary night blindness (CSNB) | - | - | - | + |
| **13** | 12 | 0.4 | VI | Infantile nystagmus syndrome | - | - | - | + |
| **14** | 7 | 0.1 | VI | Infantile nystagmus syndrome | - | - | - | + |
| **15** | 6 | 0.6 | VI | Albinism | - | - | +/- | + |
| **16** | 11 | 0.2 | VI | Macular atrophy | - | - | - | - |
| **17** | 10 | 0.8 | VI | Cone-rod dystrofy | - | - | - | - |
| **18** | 6 | 0.1 | VI | Hypermetropia | - | - | + | + |
| **19** | 8 | 0.1 | VI | Albinism | - | - | + | - |
| **20** | 7 | 0.3 | VI | Albinism | - | - | + | +/- |
| **21** | 11 | 0.5 | VI | Albinism | - | - | - | + |
| **22** | 7 | 0.4 | VI | Cone dysfunction (Bornholm) | - | - | +/- | - |
| **23** | 6 | 0.2 | VI | Coloboma of the iris and retina, and optic nerve | - | - | + | - |
| **24** | 10 | 0.4 | VI | Albinism | - | - | +/- | + |
| **25** | 9 | 0.6 | VI | Aniridia | - | - | + | + |

| Pp | Age | VA | Group | Diagnosis | Motor imp | Mental delay | Strabismus | Nystagmus |
|---|---|---|---|---|---|---|---|---|
| **26** | 12 | 0.5 | VI | Albinism | - | - | + | + |
| **27** | 11 | 0.3 | VI | Infantile nystagmus syndrome | - | - | - | + |
| **28** | 7 | 0.7 | VI | Congenital stationary night blindness (CSNB) | - | - | + | + |
| **29** | 12 | 0.3 | VI | Congenital ptosis | + | - | - | - |
| **30** | 8 | 0.2 | VI | Hypermetropia | +/- | + | - | - |
| **31** | 8 | 0.3 | VI | Hypermetropia with astigmatism | - | - | - | - |
| **32** | 14 | 0.4 | VI | Infantile nystagmus syndrome | - | - | - | + |
| **33** | 6 | 0.5 | VI | Hypermetropia with astigmatism | - | - | - | + |
| **34** | 9 | 0.5 | CVI | Noonan syndrome | - | - | + | +/- |
| **35** | 9 | 0.3 | CVI | Noonan syndrome | + | - | +/- | +/- |
| **36** | 11 | 1.0 | CVI | Status after meningitis and cerebritis | - | + | - | - |
| **37** | 7 | 0.3 | CVI | Premature | - | + | + | - |
| **38** | 10 | 0.7 | CVI | Premature | - | - | - | - |
| **39** | 8 | 0.3 | CVI | Optic nerve atrophy, microcephalus and bilateral occipital infarcts | - | - | + | +/- |
| **40** | 7 | 0.1 | CVI | Premature and dysmature | + | - | + | - |
| **41** | 8 | 0 | CVI | Joubert syndrome | - | - | + | - |
| **42** | 9 | -0.2 | CVI | Cerebral arteriovenous malformation, resulting in 2 strokes | - | - | - | - |
| **43** | 8 | -0.2 | CVI | Premature | - | + | - | - |
| **44** | 11 | 0.3 | CVI | White matter damage due to mitochondrial disease and internuclear ophthalmoplegia | + | - | + | + |
| **45** | 8 | 0 | CVI | Perinatal complications, Stickler syndrome | - | - | - | - |
| **46** | 8 | -0.1 | CVI | Premature and perinatal complications | + | - | + | - |
| **47** | 10 | 0 | CVI | Premature | + | - | - | - |
| **48** | 8 | 0.1 | CVI | Perinatal complications | - | + | + | - |

| Pp | Age | VA | Group | Diagnosis | Motor imp | Mental delay | Strabismus | Nystagmus |
|---|---|---|---|---|---|---|---|---|
| **49** | 7 | 0.3 | CVI | Dysmature, partial cataract | - | - | - | - |
| **50** | 10 | 0.1 | CVI | Exact cause unknown | + | + | - | - |
| **51** | 7 | -0.1 | CVI | Exact cause unknown | + | - | - | - |
| **52** | 11 | 0 | CVI | High-energy trauma | - | - | - | - |
| **53** | 8 | 0 | CVI | Neurofibromatosis 1 | - | - | - | - |
| **54** | 8 | 0.2 | CVI | Premature and perinatal complications | - | - | - | - |
| **55** | 6 | 0.2 | CVI | Cerebral palsy | + | - | - | +/- |
| **56** | 11 | 0.2 | CVI | High-energy trauma | - | - | - | + |
| **57** | 8 | 0.2 | CVI | Exact cause unknown | - | - | - | - |
| **58** | 9 | 0.1 | CVI | KDM5C-syndrome | - | + | - | - |
| **59** | 9 | 0.1 | CVI | Perinatal asphyxia | - | + | - | +/- |
| **60** | 8 | 0.2 | CVI | Chromosomal deletion | + | + | - | +/- |
| **61** | 13 | -0.1 | CVI | Perinatal asphyxia | - | + | - | - |
| **62** | 9 | 0.1 | CVI | Cerebral palsy | - | + | - | - |
| **63** | 7 | 0.3 | CVI | Bosch-Boonstra-Schaaf optic atrophy syndrome (BBSOAS) | + | - | - | - |
